# Effects of Lithium Slag on the Frost Resistance of Cement-Soil

**DOI:** 10.3390/ma15165531

**Published:** 2022-08-11

**Authors:** Zhi Chen, Sili Chen, Liwen Liu, Yuwan Zhou

**Affiliations:** School of Architecture and Civil Engineering, Shenyang University of Technology, Shenyang 110870, China

**Keywords:** cement-soil, lithium slag (LS), frost resistance, unconfined compression strength, triaxial compression strength, nuclear magnetic resonance (NMR), scanning electron microscope (SEM)

## Abstract

In this study, the effect of lithium slag (LS) on the frost resistance of cement-soil was evaluated. The results of freeze–thaw damage on the surface of the cement-soil, freeze–thaw mass loss, unconfined compression strength, triaxial shear strength, cohesion, and internal friction angle were tested at various freeze–thaw cycles after 90 days of curing when LS was incorporated into the cement-soil at different proportions (0%, 6%, 12%, and 18%). Combining nuclear magnetic resonance (NMR) T_2_ distribution and scanning electron microscopy (SEM) microscopic images, the mechanism of the effect of LS on the cement-soil was also analyzed. The experiment confirmed that the surface freeze–thaw damage degree and mass loss value of the cement-soil decreased after incorporating different LS contents, and that the unconfined compression strength, triaxial shear strength, cohesion, and internal friction angle also improved significantly compared with the specimens without LS. In this experiment, the optimization level of the cement-soil performance with different LS content was ranked as 12% > 18% > 6% > 0%. According to the NMR and SEM analysis results, the LS content of 12% can optimize the internal pore structure of the cement-soil and strengthen the bond between aggregate particles, hence inhibiting the extension of freeze-swelling cracks induced by freeze–thaw cycles. In conclusion, LS can effectively enhance the frost resistance of cement-soil, and the optimum content in this experiment is 12%.

## 1. Introduction

Cement-soil is a material made by mixing cement and soil with water in a certain ratio [1,2]. It can be used as the primary material in channel seepage control, dam slope protection, and road stabilization layer owing to the convenience with which it can be prepared and obtained from local materials [3,4,5,6,7]. However, in northern regions of China, where the temperature difference between day and night is great, repeated freezing and sun exposure pose a significant threat to the performance stability of cement-soil [8,9]. Owing to the frequent alternation between low and normal temperatures, it is very easy for crack expansion and pore enlargement to occur within the cement-soil material, thereby threatening the structure’s stable service [10,11].

In recent years, many researchers have conducted many studies to improve the frost resistance of cement-soil materials [8,12,13]. In order to promote resource utilization and reduce carbon emissions, researchers often mix some suitable solid wastes into cement-soil for exploration, including ferronickel slag powder, rubber powder, straw, pumice powder, rice husk ash, and so on [2,8,14,15,16,17]. Wang et al. [12] investigated the effect of incorporating rubber powder into the cement-soil on its frost resistance and found that the incorporation of rubber powder improved the bonding skeleton within the cement-soil, which could effectively improve the frost swelling effect of the cement-soil affected by freeze–thaw cycles and inhibit the crack expansion of the cement-soil under the freeze–thaw environment, thus improving the overall frost resistance of the cement-soil. Zhang et al. [18] evaluated the effect of fly ash and cement to stabilize the frost resistance of loess with the objective of improving the freeze–thaw durability of loess and discovered, through three experiments of unconfined compression strength tests, freeze–thaw durability tests, and frost-heave and thaw-weakening tests, that mixing fly ash in loess can improve the freeze–thaw performance of loess, but the enhancement effect is very limited, whereas mixing cement with loess can have a better curing effect, which can effectively prevent the external water from entering into the soil to completely weaken its freeze swell during freeze–thaw. Xu and Niu [19] studied the effect of polypropylene fibers on the freeze–thaw performance of the cement-soil, and the experimental results showed that mixing the cement-soil with an appropriate content of polypropylene fibers could effectively improve the strength and freeze–thaw durability, and the most significant effect on the performance of the cement-soil was achieved when the content of polypropylene fibers was 0.1% and the length was 3 mm, which was primarily because of the fact that the polypropylene fibers hindered the expansion of internal microfractures during the freeze–thaw process. According to the aforementioned research findings, the dense internal space structure effectively resists the growth of cracks and pores under freeze–thaw action, which is the primary reason for the improvement in the frost resistance of cement-soil. On this basis, the authors discovered that lithium slag (LS), which is widely used in the preparation of concrete materials, has a comparable function [20].

LS is an industrial waste residue produced during the production of lithium carbonate by the sulfuric acid method after high-temperature roasting of lithium pyroxene ore [21]. In the common preparation process, the production of 1 ton of lithium carbonate will generate about 9–10 tons of LS [22,23], and the extremely large amount of LS stockpile not only occupies a large amount of land resources [24], but the harmful metal ions in the LS will seep into the underground with rainwater, posing a threat to soil ecological safety and resulting in the contamination of groundwater [25,26]. Fortunately, the researchers discovered that LS is suitable as an admixture for concrete materials because of its high ${\mathrm{SiO}}_{2}$ and ${\mathrm{Al}}_{2}O_{3}$ content and high pozzolanic activity [24,27], which can contribute to the utilization of bulk solid waste and provide an important perspective for promoting the “carbon peaking and carbon neutrality” objective in cement-based materials. He et al. [28] studied the microstructure of concrete with different proportions of silica fume replaced by LS in the preparation of ultra-high performance concrete (UHPC) and revealed that, although the incorporation of LS reduced the microstructure of UHPC in the early stage, the incorporation of LS greatly improved the hydration reaction of UHPC, increased the elastic modulus at the transition interface, and enhanced the internal compactness of concrete in the later stage. He et al. [22] evaluated the effect of LS on concrete properties by incorporating LS into concrete in varying percentages, and discovered that the appropriate content of LS can lead to a better pozzolanic performance in concrete, and the secondary hydration product C-S-H can better fill the internal pores of concrete, thereby enhancing the compressive strength, elastic modulus, dry shrinkage, and creep properties of concrete. Wu et al. [29] investigated the performance of high-performance concrete (HPC) incorporating LS and fly ash, and reported that the incorporation of LS led to the consumption of $\mathrm{Ca}{\left(\mathrm{OH}\right)}_{2}$ crystals by secondary hydration reaction within the concrete, which substantially increased the mechanical properties, cracking resistance, and impermeability of HPC. Consequently, LS has contributed to the improvement in concrete performance primarily because its pozzolanic activity can cause secondary hydration reactions with cement hydration products, and the secondary hydration products with cementitious properties can effectively increase the internal bond strength and decrease the porosity of concrete.

According to the literature review, although researchers have conducted a substantial amount of research on admixtures in cement-soil as well as on the effect of LS mixed into concrete on its properties, there are few reports on LS incorporated into cement-soil. This paper tries to explore the influence of LS in the cement-soil on its frost resistance performance, and evaluates the effect of different LS contents (0%, 6%, 12%, and 18%) on the surface damage and mass loss, unconfined compression strength, triaxial shear strength, cohesion, and internal friction angle of the cement-soil at different freeze–thaw cycles (0, 2, and 4), and NMR and SEM were also utilized to analyze, from a microscopic perspective, the mechanism of the effect of LS on cement-soil. This study aims to provide ideas for improving the frost resistance of cement-soil and advancing the comprehensive utilization of LS.

## 2. Materials and Methods

### 2.1. Materials

**Figure 1:**
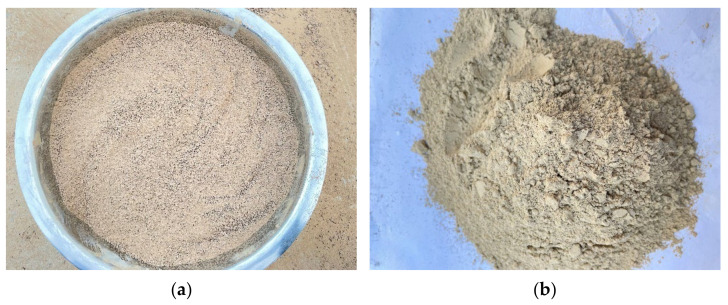
Images of the soil and LS used in the experiment: (**a**) soil and (**b**) LS.

**Figure 2:**
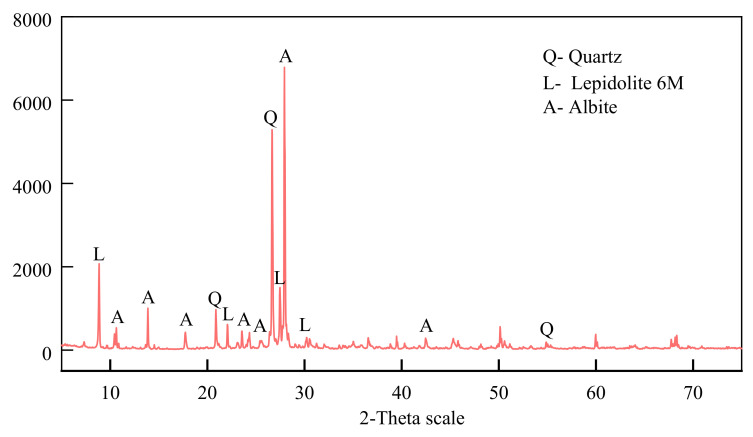
XRD pattern of LS.

The cement used in this study was conventional Portland cement P.O 42.5 from a cement factory in Liaoning province, and the cement used in this experiment was produced by the same batch. The basic properties of the cement are shown in Table 1, and the chemical composition is shown in Table 2. The testing was carried out by requirements of the national standard “Common Portland Cement” (GB175-2007). The experimental soil was taken from the silty clay in the soil layer of 2.8 m below the surface in an area of Liaoning Province, as shown in Figure 1a. Before the experiment, it was air-dried, ground, and passed through a 5 mm sieve. The physical indexes of the experimental soil are shown in Table 3, which meet the requirements of the national industrial standard “Specification for Design of Cement-Soil Mixture Ratio” (JGJ/T 233-2011). LS was taken from a factory in Shandong Province, as shown in Figure 1b. LS in this experiment was the main product discharged in large quantities after washing in the production of lithium carbonate from spodumene, which was air-dried and ground before the experiment; the particle size was 10 μm−90 μm. The chemical composition is shown in Table 2, and the X-ray diffraction (XRD) diagram is shown in Figure 2. Local tap water was used as the experimental water.

Table 2 shows that the main components of the LS applied in this experiment are ${\mathrm{SiO}}_{2}$. and ${\mathrm{Al}}_{2}O_{2}$, indicating that LS has a high pozzolanic activity [27,30]. From the XRD diffraction diagram of Figure 2, the main phases in LS contain quartz, accounting for 35.1%, which will improve the strength of the specimen to a certain degree [24].

### 2.2. Mix Proportion

In accordance with the national industrial standards of “Specification for Mix Proportion Design of Cement-Soil” (JGJ/T 233-2011), this experiment set the water–binder ratio of 1.7; the cement content of 12%; and the four control groups with LS contents of 0%, 6%, 12%, and 18%. The specimen size was 39.1 mm for the inner diameter and 80 mm for the height. The specimens of each group were formulated as shown in Table 4.

### 2.3. Preparation Process

**Figure 3:**
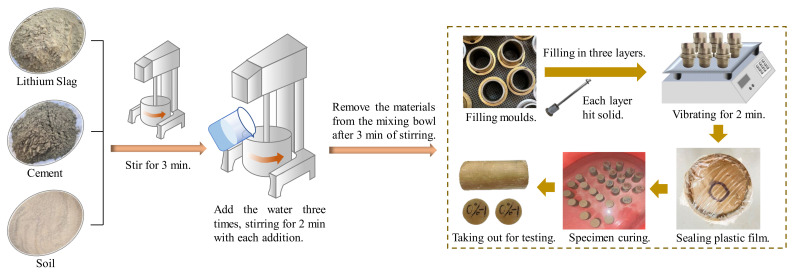
The preparation flow chart of test specimens.

The process of specimen preparation in this experiment is shown in Figure 3. The process involves mixing the LS, cement, and soil for three minutes in a planetary mixer, then adding the water three times, each time stirring for two minutes before adding the next addition, and then stirring for three minutes after all of the water has been added. Before filling the mold, the interior of the mold was coated with vaseline to facilitate demolding. The cement-soil mold was filled in three layers, with each layer being struck 15 times with a ramming hammer to ensure uniform density and thickness. Before filling the subsequent layer, the surface of the previous layer was lightly scraped to ensure adequate adhesion. The specimen was placed on the shaking table after filling and vibrated for two minutes to make the cement-soil mixture dense. Following the completion of the vibration process, the surface was smoothed and sealed with plastic film. After 24 h at room temperature, the sample was demolded, weighed, numbered, and cured in water. The curing water was 20 mm above the specimen’s surface. The specimen was removed after 90 days of curing, and the experiment was conducted immediately after drying.

### 2.4. Test Methods

#### 2.4.1. Freeze–Thaw Cycles Test

The TDR-10 rapid freeze–thaw instrument was used for freeze–thaw experiments, the freezing temperature was set to −15 °C, the thawing temperature was set to 20 °C, the freezing and thawing time was 12 h, and it took 24 h to complete one freeze–thaw cycle. Corresponding experiments were carried out after two and four freeze–thaw cycles. The test result was taken as the average value of three test specimens.

#### 2.4.2. Unconfined Compression Strength Test

The WDW-100E universal pressure tester was adopted to carry out the unconfined compression strength test. The experimental steps followed the relevant requirements of the “Specification for Mix Proportion Design of Cement-Soil” (JGJ/T 233-2011), and the loading rate was set to 0.8 mm/min. The cement-soil specimens after 90 days of curing were taken out and placed in a ventilated area to dry until the weight of the specimens was the same as the weight at the time of demoulding, and then the experiment was carried out. The test result was taken as the average value of three test specimens.

#### 2.4.3. Triaxial Compression Test

The TCK-1 triaxial test measuring control instrument and TSZ-6 strain-controlled triaxial instrument were used to conduct the experiment, and the experimental procedure followed the relevant requirements of the “Specification for Mix Proportion Design of Cement-Soil” (JGJ/T 233-2011) and “Standard for Soil Test Method” (GB/T 50123-1999). The cement-soil specimens were taken out and air-dried until the quality was the same as the sample preparation, and then the experiment was conducted with the unconsolidated undrained compression (UU) test method; the confining pressures were 0.3 MPa, 0.6 MPa, and 0.9 MPa, respectively. The test result was taken as the average value of three test specimens.

#### 2.4.4. NMR Test

The MesoMR core nuclear magnetic resonance imaging analyzer with Newmark NMR analysis application software V4.0 was applied for the NMR experiments, and the cement-soil specimens after 90 days of curing were subjected to comparative T_2_ mapping analysis.

#### 2.4.5. SEM Test

The Hitachi Regulus 8100 scanning electron microscope was used. The microstructure of cement-soil specimens after 90 days of curing was compared and analyzed.

## 3. Results and Discussion

### 3.1. Frost Resistance Analysis

The surface morphology of specimens after freeze–thaw cycles can intuitively reflect the effect of the LS content on the frost resistance of cement-soil. At 0, 2, and 4 freeze–thaw cycles, the surface morphology of each group of specimens cured for 90 days was compared, and the mass loss of each group after freeze–thaw cycles was counted. The average mass loss value of the three specimens was taken.

**Figure 4:**
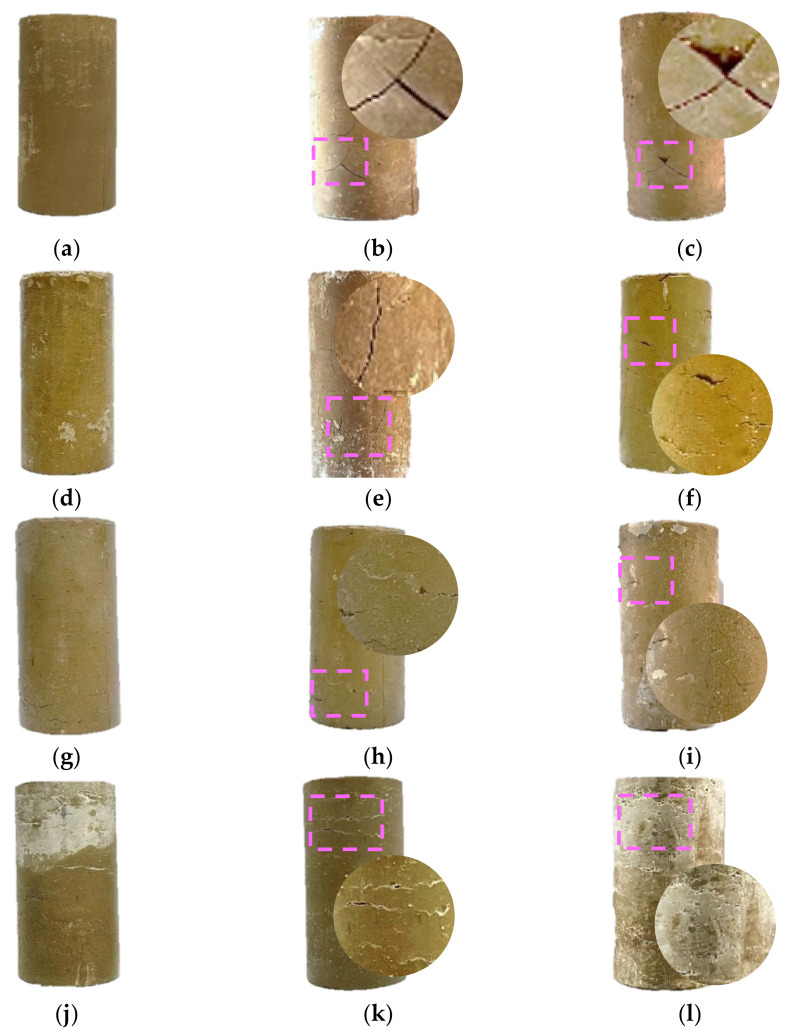
Surface damage of each group of specimens before and after freeze–thaw cycles: (**a**) LC-0 specimen group with 0 freeze–thaw cycles; (**b**) LC-0 specimen group with 2 freeze–thaw cycles; (**c**) LC-0 specimen group with 4 freeze–thaw cycles; (**d**) LC-6 specimen group with 0 freeze–thaw cycles; (**e**) LC-6 specimen group with 2 freeze–thaw cycles; (**f**) LC-6 specimen group with 4 freeze–thaw cycles; (**g**) LC-12 specimen group with 0 freeze–thaw cycles; (**h**) LC-12 specimen group with 2 freeze–thaw cycles; (**i**) LC-12 specimen group with 4 freeze–thaw cycles; (**j**) LC-18 specimen group with 0 freeze–thaw cycles; (**k**) LC-18 specimen group with 2 freeze–thaw cycles; (**l**) LC-18 specimen group with 4 freeze–thaw cycles.

The comparison of surface morphology of each group before and after freeze–thaw cycles is shown in Figure 4. It can be observed that the specimens of the LC-0 group without LS have clear surface deep cracks after 2 and 4 freeze–thaw cycles, and the damage of freeze–thaw is very obvious, whereas the specimens of LS have varying degrees of improvement in surface damage after freeze–thaw compared with the LC-0 group, and the surface damage cracks, as well as the scope and depth of damage, were reduced. It is noteworthy that the LC-12 group with a 12% LS content has the least surface damage after 2 and 4 freeze–thaw cycles, and its surface integrity is the best when compared with other groups. This shows that the incorporation of LS can improve the frost resistance of the cement-soil to a certain extent, and the incorporation of 12% LS can achieve the best frost resistance of the cement-soil in terms of the degree of surface freeze–thaw damage.

The mass loss values of each specimen group after freeze–thaw cycles were calculated according to Equation (1):

(1) $$\Delta m_{n}=\frac{m_{0}-m_{n}}{m_{0}}\times 100\%$$

where $\Delta m_{n}$ is the mass loss rate, $m_{0}$ is the initial mass of the specimen, and $m_{n}$ is the mass of the specimen after *n* freeze–thaw cycles (*n* = 2, 4).

**Figure 5:**
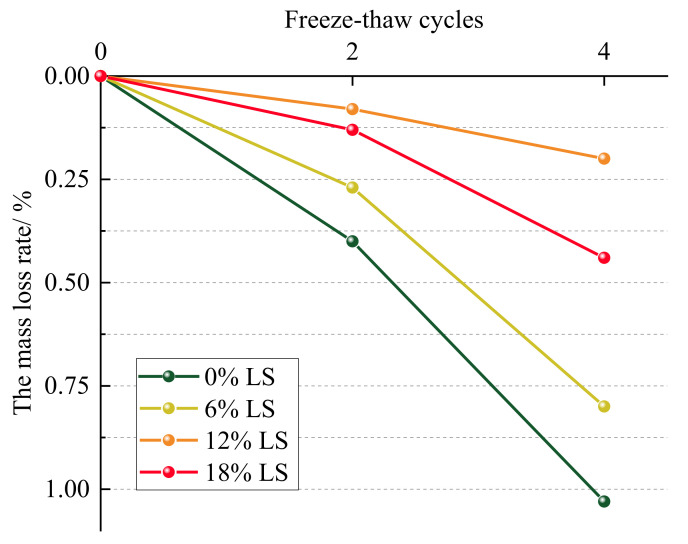
Effect of LS content on mass loss of cement-soil after freeze–thaw cycles.

Figure 5 illustrates the mass loss of each group of specimens before and after freeze–thaw cycles, and it can be seen that the mass loss rate of each group of specimens increases as the number of freeze–thaw cycles increases. According to Figure 5, the specimen of the LC-0 group has the greatest mass loss rate after 4 freeze–thaw cycles, with a mass loss rate of 1.03%. However, the incorporation of LS has reduced the mass loss rate of the specimen after freeze–thaw cycles, with the LC-12 group having the smallest mass loss rate, with a loss rate of 0.20%. After 4 freeze–thaw cycles, the mass loss rate of the specimen groups with 6% and 18% LS is 0.80% and 0.44%, respectively, which is less than that of the LC-0 group, indicating that the improvement of the frost resistance performance of the cement-soil varied with the dosing of LS. In terms of surface damage and mass loss, the optimal level of frost resistance of cement-soil with different contents of LS is 12% > 18% > 6% > 0%.

From the surface damage and mass loss of the specimens after freeze–thaw cycles in each group, it is evident that the incorporation of LS improved the frost resistance of the cement-soil, and this result is similar to the previous studies that investigated the effect of micro-LS on the frost resistance of high-performance concrete [29]. This is primarily because of the fact that the LS applied in this experiment contains more ${\mathrm{SiO}}_{2}$ and ${\mathrm{Al}}_{2}O_{3}$, which have high pozzolanic activity and can react with $\mathrm{Ca}{\left(\mathrm{OH}\right)}_{2}$ produced by cement hydration to produce hydrated calcium silicate gel and hydrated calcium aluminate [30], thereby enhancing the compactness of the cement-soil and decreasing the internal microcracks, reducing the risk of microcrack penetration during freezing, and effectively resisting crack growth and specimen mass loss after freeze–thaw cycles [11].

### 3.2. Unconfined Compression Strength Ansysis

**Figure 6:**
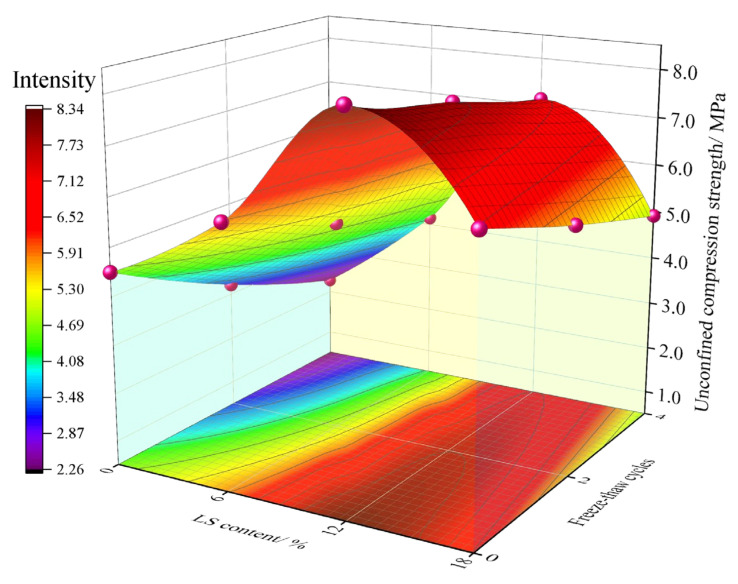
Effect of LS content on unconfined compression strength of cement-soil after freeze–thaw cycles.

The unconfined compression strength of each group of specimens cured for 90 days was tested for 0, 2, and 4 freeze–thaw cycles, respectively. The unconfined compression strength values of each group of specimens are shown in Figure 6.

Figure 6 presents that incorporating LS significantly improves cement-soil’s unconfined compression strength. Consistent with what can be seen in Figure 5, the ranking of the effect of different LS content incorporation to improve the unconfined compression strength still follows 12% > 18% > 6% > 0%. The unconfined compression strength values of the LC-12 group with 12% LS incorporation are the highest in the same category for 0, 2, and 4 freeze–thaw cycles, respectively, with the greatest strength values being 8.33 MPa, 7.72 MPa, and 7.13 MPa, respectively, which are 3.83 MPa, 4.51 MPa, and 4.85 MPa higher, respectively, than those of the LC-0 group without LS. From the dense clouds in Figure 6, it can be seen that the density of the clouds corresponding to the specimen group with 0% LS content is low at 0, 2, and 4 freeze–thaw cycles, indicating that the unconfined compression strength value decreases significantly after freeze–thaw cycles, whereas the density of the clouds corresponding to the specimen group with 12% LS content is large at 0, 2, and 4 freeze–thaw cycles, and the specimen groups with 0%, 2%, and 4% LS content are also larger than those of the specimen group without LS; this reflects that the decline degree of the unconfined compression strength value after freeze–thaw cycles was less than that of the specimen group without LS. This proves that the incorporation of LS can improve the unconfined compression strength of the cement-soil, reaffirming that LS can improve the frost resistance performance of the cement-soil, as described in Section 3.1, and demonstrates that an LS content of 12% can achieve the best performance of the cement-soil.

The analysis of the cause is consistent with the description in Section 3.1. It is primarily due to the reaction of the higher activity of ${\mathrm{SiO}}_{2}$ and ${\mathrm{Al}}_{2}O_{3}$ in LS with cement hydration products to generate C-S-H with cementitious properties, which can effectively increase the bond strength between particles within the cement-soil and reduce the porosity to form a denser particle space [24], thereby increasing the unconfined compression strength of the cement-soil. Park et al. [31] proposed that oyster-shell powder (OSP)+ steelmaking slag dust (SMS) could have a more active pozzolanic reaction when studying the influence of industrial by-products on the unconfined compression strength of solidified organic marine clay soils, so as to improve the compressive strength of compressive cement-soil; Shi et al. [32] investigated the incorporation of steel slag into dredged silty, and pointed out that the pozzolanic activity of steel slag could further react with cement hydration products to generate more cementitious materials and then fill the internal pores of soil. These improvement principles are consistent with the conclusions of this paper. It is worth mentioning that the content of LS has an optimal range in this experiment, and when the content of LS is larger (18%), the frost resistance and unconfined compression strength improvement effect of the cement-soil are reduced; this phenomenon is similar to the experimental results of Shi et al. [33] and He et al. [22]. This is mainly because of the excessive content of LS, which accelerates the rapid consumption of cement hydration products and relatively reduces the LS content involved in the reaction, thereby reducing the potential of improving the cement-soil performance [34].

### 3.3. Triaxial Shear Test Ansysis

#### 3.3.1. Stress–Strain Curve

**Figure 7:**
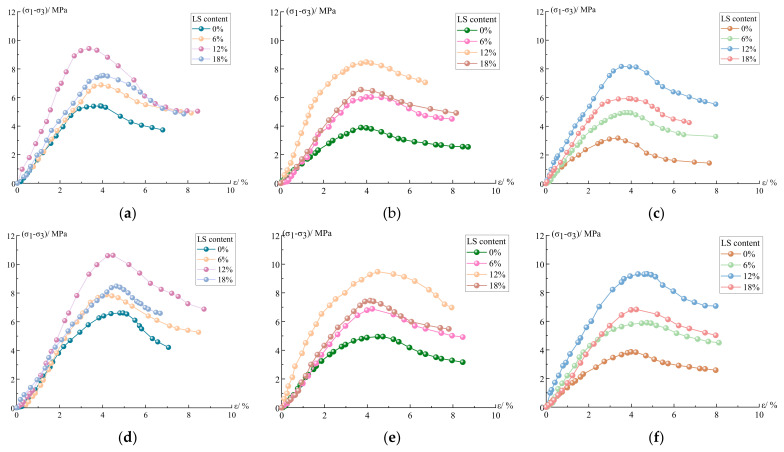

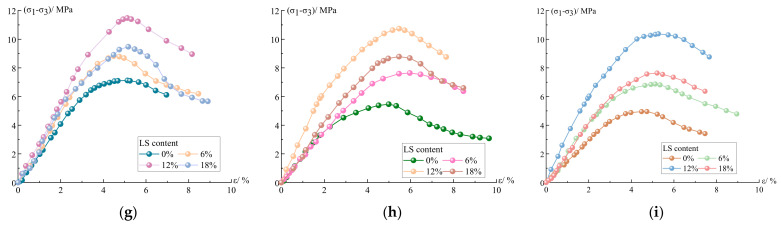
Triaxial compressive stress–strain curve: (**a**) 0 freeze–thaw cycles under 0.3 MPa confining pressure; (**b**) 2 freeze–thaw cycles under 0.3 MPa confining pressure; (**c**) 4 freeze–thaw cycles under 0.3 MPa confining pressure; (**d**) 0 freeze–thaw cycles under 0.6 MPa confining pressure; (**e**) 2 freeze–thaw cycles under 0.6 MPa confining pressure; (**f**) 4 freeze–thaw cycles under 0.6 MPa confining pressure; (**g**) 0 freeze–thaw cycles under 0.9 MPa confining pressure; (**h**) 2 freeze–thaw cycles under 0.9 MPa confining pressure; (**i**) 4 freeze–thaw cycles under 0.9 MPa confining pressure.

According to the cement-soil experiment by Wang et al. [35], the stress–strain curve can be divided into five stages: compaction stage, linear elastic stage, plastic stage, softening stage, and residual strength stage. The stress–strain curves for each group of specimens in this experiment under different confining pressures are presented in Figure 7, and it can be seen that the stress–strain curves obtained in this experiment have the same characteristics as those derived by Long et al. [36] and Zhang et al. [37]. Analyzing the characteristics of the stress–strain curves in each stage of the experiment, the curve of the compaction stage is slightly concave, because the contact surface of the specimen and the compression plate are not completely closed, and the internal part of the cement-soil is not completely dense, so the process of gap compacting will occur first under the action of pressurization. The curve of the elastic stage is straight and the stress–strain relationship is approximately linear, thus the specimen is deformed elastically during the process of pressurization and the elastic modulus is at a constant value. The pores and cracks inside the specimen develop rapidly in the plastic stage, and the rate of increase in strain is greater than that in stress, and the peak of stress appears. The curve of the softening stage shows a decreasing trend, and the stress value decreases continuously after the peak and decreases with the increase in strain. The curve of the residual strength stage is approximately horizontal, the stress value remains unchanged, and the strain value increases continuously; at this time, the load-bearing capacity of the specimen is not completely lost, but has a certain residual strength.

From the trend presented in the curve of Figure 7, the ascending section of the stress–strain curve of the specimens doped with LS generally moves to the left; its slope and peak stress increase and peak strain decreases, indicating that the doping of LS can effectively improve the elastic modulus of the cement-soil specimens, and He et al. [28] mentioned that LS can improve the elastic modulus of concrete in the study of adding LS into UHPC, which is similar to the results of this study. Under three different confining pressures, the peak stress and peak strain of the curve increased as the confining pressures increased, and the decline section of the curve has a slowing trend with the increase in confining pressure. Nasrollahzadeh and Nouhi [38] have explained this phenomenon, pointing to the fact that the dopant can increase the specimen’s lateral stiffness, necessitating greater pressure during the specimen’s compressive testing, and because of the increased lateral stiffness, which limits the specimen’s compressive expansion and thus reduces the axial strain due to Poisson’s effect.

**Figure 8:**
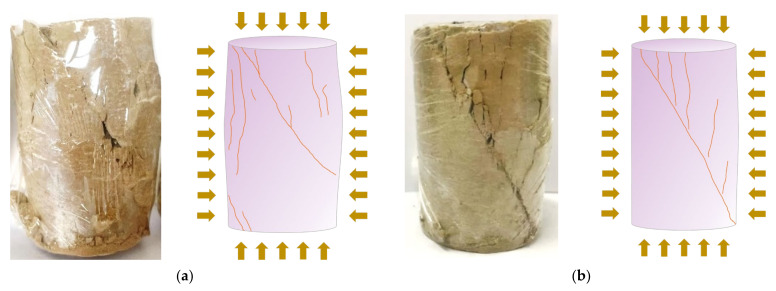
The failure of two specimens under confining pressure of 0.9 MPa: (**a**) LC-0 specimen group and (**b**) LC-12 specimen group.

Without considering the freeze–thaw cycles’ effect, it is obvious from Figure 7a,d,g that the incorporation of LS has a significant improvement effect on the peak strength of the cement-soil, and that the improvement effect is greatest at 12% LS incorporation. This proves that the incorporation of LS also has a significant effect on the enhancement of the strength of the cement-soil, which is similar to the phenomenon proposed by Nasrollahzadeh and Nouhi, where the incorporation of LS into cement-soil in this experiment can improve the lateral stiffness of the cement-soil specimens to some extent, resulting in an increase in peak stress and a decrease in axial strain in the cement-soil specimens. This mechanism is also the reason that LS guarantees an increase in the frost resistance of cement-soil. Figure 8 shows the failure pattern of LC-0 specimens without LS and LC-12 specimens with 12% LS in triaxial shear test under 0.9 MPa confining pressure. It can be seen that the damage mode of specimens without LS exhibits obvious bulging, significant shear damage characteristics, splitting damage at the bottom and local surface of the specimens, and severe overall collapse, while the failure pattern of specimens with 12% LS displays slight swelling, obvious shear damage characteristics, local splitting damage, and less overall collapse, indicating that LS can increase the elastic modulus of cement-soil and enhance the brittleness.

**Figure 9:**
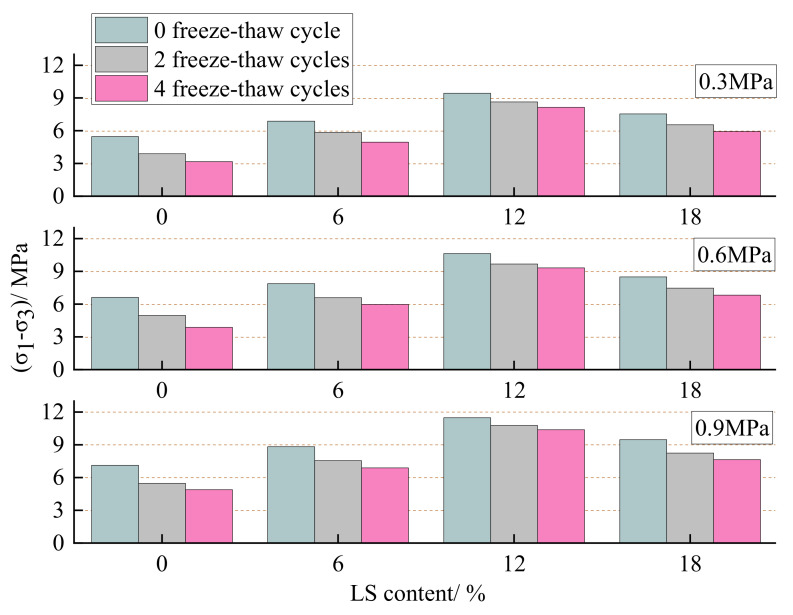
Effect of LS content on unconfined compression strength of cement-soil after freeze–thaw cycles.

Considering the effect of freeze and thaw, it is also apparent that the peak stress of the LC-0 group without LS decreases significantly under the three confining pressures, while the peak stress decrease of the specimens with LS is less than that of the LC-0 group, and the peak stress decrease of the LC-12 group with 12 percent LS is the smallest. At the confining pressure of 0.3 MPa, the peak stress of the specimens in the LC-0 group without LS doping decreased by 41.1% after 4 freeze–thaw cycles, while the peak stress of the specimens in the LC-12 group with 12% LS content decreased by only 13.4%; at 0.6 MPa, the peak stress of the specimens in the LC-0 group decreased by 40.9%, while the peak stress of the LC-12 group decreased by only 12.4%; at 0.9 MPa, the peak stress of LC-0 group specimens decreased by 30.4%, while the peak stress of LC-12 group specimens decreased by only 9.6%. Figure 9 can more intuitively illustrate the influence of different LS content on the frost resistance of cement-soil under varying confining pressures. Under three confining pressures, the improvement effect of LS on the frost resistance of cement-soil is still in line with 12% > 18% > 6% > 0%, revealing that LS can not only improve the peak strength of the cement-soil specimens, but also boost the frost resistance, and the performance improvement is greatest when the LS content is 12%, which is consistent with the experimental results in Section 3.1 and Section 3.2.

Its improved performance is consistent with that described in the previous section, primarily because the chemical composition of LS has a certain reaction activity, which can react with the hydration products produced by cement hydration in the process of cement-soil maintenance to produce filled particles with cementitious properties, which not only increases the bonding strength between soil particles, but also significantly reduces the internal voids of the cement-soil; this is more similar to the application mechanism in coastal soil modified with cement and fly ash by Li et al. [39]. Tan et al. [40] has reported that the reaction product of high pozzolanic activity of micro-LS can optimize the pore structure, and thereby enhance the performance of cement when studying the preparation of micro-LS via wet grinding and its application as an accelerator in Portland cement. Li and Xia [20] also pointed out that LS can react with $\mathrm{Ca}{\left(\mathrm{OH}\right)}_{2}$ to generate C-S-H and calcium aluminate hydrate, which can fill the pores of concrete and strengthen the internal compactness, thus improving the performance of concrete. Therefore, the compact and dense internal structure is an important reason for the improvement of the cement-soil performance.

#### 3.3.2. Cohesion and Internal Friction Angle

**Figure 10:**
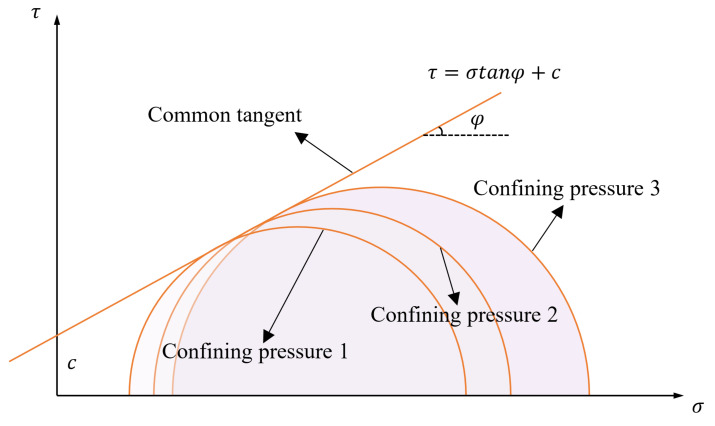
Effect of LS content on unconfined compression strength of cement-soil after freeze–thaw cycles.

According to Figure 8, showing the stress–strain curve, the Mohr circles of stresses at 0.3 MPa, 0.6 MPa, and 0.9 MPa are plotted with $\frac{\sigma_{1}+\sigma_{3}}{2}$ as the center and $\frac{\sigma_{1}+\sigma_{3}}{2}-\sigma_{3}$ as the radius, and the intersection points of the Mohr circles with the x-axis are $\sigma_{1}$ and $\sigma_{3}$, respectively. The common tangent of the stress circles under the three kinds of confining pressures is the shear strength envelope, and the cohesion c and internal friction angle φ of each specimen are obtained. The schematic diagram of the molar stress circle and shear strength envelope is shown in Figure 10.

**Figure 11:**
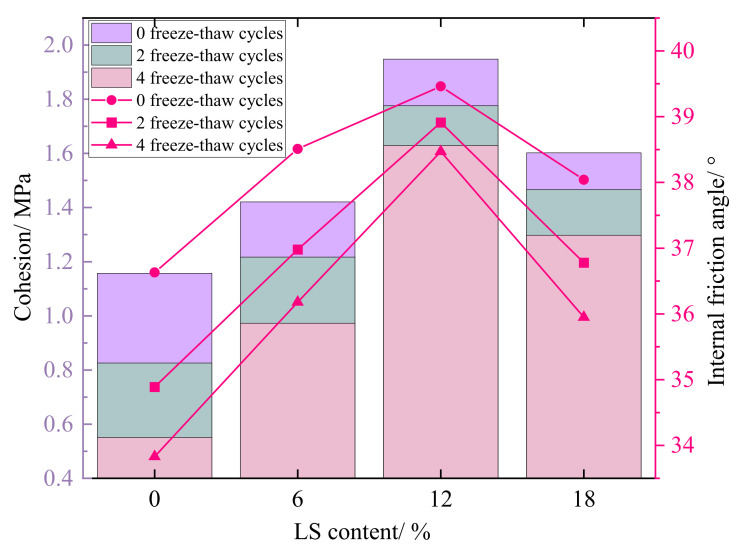
Effect of LS content on unconfined compression strength of cement-soil after freeze–thaw cycles.

The relationship between the cohesion c and internal friction angle φ of each group of specimens before and after freeze–thaw and the amount of LS is shown in Figure 11. The cohesion and internal friction angle are improved to different degrees after the incorporation of LS into the cement-soil, and the cohesion of the specimen in the LC-0 group without LS is 1.16 MPa under 0 freeze–thaw cycles, while the cohesion of the cement-soil with 6%, 12%, and 18% of LS is increased by 22.8%, 68.3%, and 38.5%, respectively, compared with the LC-0 group. The improvement effect of the internal friction angle is correlated with the LS content, which is 39.5° at 12% LS content, a slight increase from 36.6° in the LC-0 group. Under the action of freeze–thaw cycles, the effect of LS on the improvement of cohesion and internal friction angle are governed by the same law. When the content of LS is 0%, freeze–thaw cycles have a significant impact on the cohesion, which decreases by 0.33 MPa after 2 cycles and 0.28 MPa after 4 cycles, but the decrease in cohesion of the specimen group mixed with LS after freeze–thaw cycles is less than that of the LC-0 group without LS. Similarly, when the content of LS is 12%, the cohesion has the smallest loss under freeze–thaw action, decreasing by 0.17 MPa after 2 freeze–thaw cycles and 0.15 MPa after 4 freeze–thaw cycles. For the LC-0 group specimens, the internal friction angle was affected by freeze–thaw cycles in the range of 36.6°–33.8°, whereas the range of change for the LC-12 group specimens with 12% LS was 39.5°–38.5°, indicating that 12% LS content in the cement-soil also improves the internal friction angle obviously.

The cohesion and internal friction angle of the cement-soil are the main factors affecting the shear performance, and the above results all indicate that the incorporation of LS can improve the cohesion and internal friction angle of the cement-soil, with the enhancement effect on the cohesion being more noticeable, also confirming the enhancement law of LS on the shear strength of the cement-soil shown in Figure 9. The experimental results are consistent with the test results of Zhao et al. [41] on the shear strength characteristics of cement-improved soil under freeze–thaw cycles.

### 3.4. Microscopic Analysis

According to the results described in the previous section, the incorporation of LS can improve the performance of cement-soil to differing degrees. Based on the results of this experiment, the 12% LS incorporation rate can maximize the performance of cement-soil. In order to analyze the improvement mechanism of LS on the performance of cement-soil from a micro perspective, the LC-0 group and LC-12 group specimens after 90 days of curing were compared and analyzed, including NMR T_2_ distribution analysis and SEM analysis.

#### 3.4.1. NMR T_2_ Distribution Analysis

**Figure 12:**
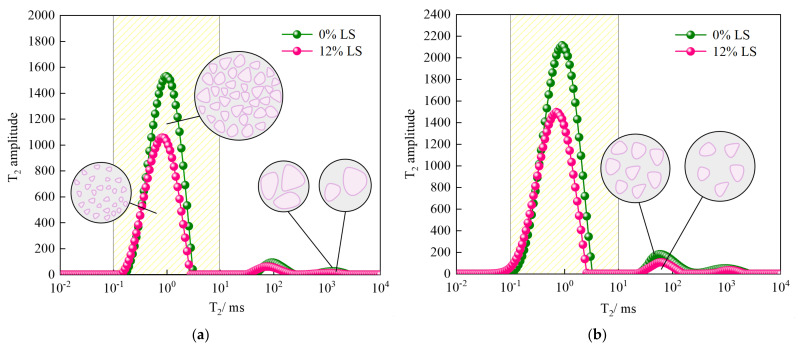
T_2_ distributions of cement soil with freeze–thaw cycles times of 0 and 4 at different LS contents: (**a**) 0 freeze–thaw cycles and (**b**) 4 freeze–thaw cycles.

The transverse relaxation time reflects the free degree of pore water in the specimen; the shorter the time, the closer the combination of water and material [42,43]. In the saturated state, the value of T_2_ can reflect the size of the internal pores of the specimen, and the smaller the T_2_ value, the smaller the pore size; the amplitude of T_2_ can reflect the number of internal pores, and the larger the value, the greater the amount of pores at that size [44,45]. Figure 12 shows the T_2_ distribution of LC-0 and LC-12 group specimens without freeze–thaw and after 4 freeze–thaw cycles. It can be seen that the T_2_ value corresponding to the maximum amplitude is in the range of 0.1–10 ms, indicating that the majority of the specimen’s interior consists of micro-pores, and that the T_2_ value has a micro-peak around 100 ms and 1000 ms, indicating that the specimen also contains mesopores and macropores. By incorporating 12% LS into the specimens, the T_2_ peak value was significantly reduced when the specimens were not frozen–thawed. The overall curve was below the curve of the specimen group without LS, and the curve of the specimen group with 12% LS shifted to the left compared with the curve of the specimen group without LS, indicating that the incorporation of LS significantly reduced the number of pores and the pore size in the specimen. Similarly, the T_2_ curves of the two groups of specimens after 4 freeze–thaw cycles also have the same law, but the three groups of T_2_ peak values of the two groups of specimens both increased, indicating that the micropores, mesopores, and macropores inside the specimens affected by freeze–thaw cycles have increased, and it evident that the addition of LS greatly reduces the internal pores of the specimen after freeze–thaw cycles. The pore size is also smaller than that of the specimen without LS, which is the key to improving the performance of cement-soil.

#### 3.4.2. SEM Analysis

**Figure 13:**
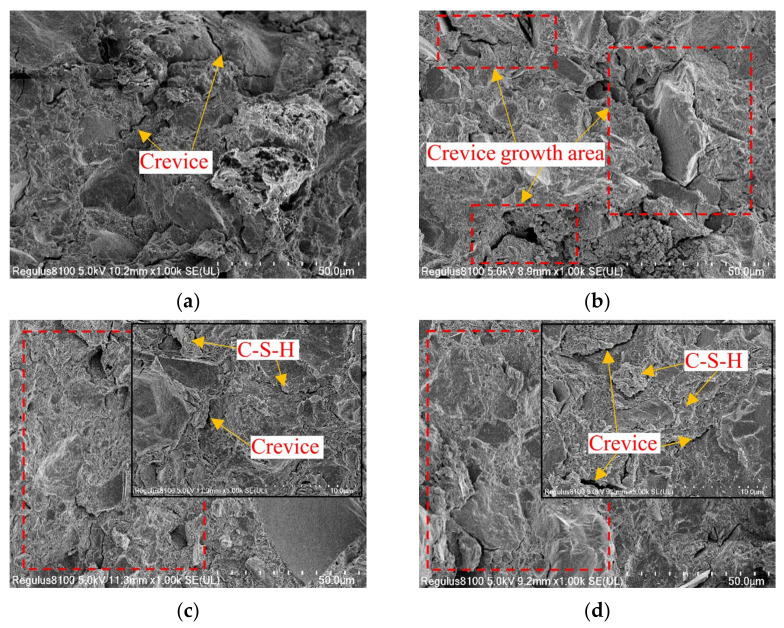
SEM images of LC-0 specimens and LC-12 specimens: (**a**) 0 freeze–thaw cycles of LC-0 specimens; (**b**) 4 freeze–thaw cycles of LC-0 specimens; (**c**) 0 freeze–thaw cycles of LC-12 specimens; (**d**) 4 freeze–thaw cycles of LC-12 specimens.

In Section 3.4.1, the effect of LS incorporation on the internal pore structure of the cement-soil was analyzed, whereas SEM scanning of specimen slices can more intuitively reflect the internal pore morphology of the cement-soil. Similarly, the 12% LS doping in this paper can lead to the greatest improvement in the performance of the cement-soil, so the two groups of specimens with 0% and 12% LS doping continued to be analyzed by SEM comparison. Figure 13 shows the SEM images of the specimens of the LC-0 group and LC-12 group with 0 freeze–thaw cycles and after 4 freeze–thaw cycles, respectively. As shown in Figure 13a,b, when the LS content is 0%, some deep cracks can be observed in the interior without freeze–thaw cycles, and the structure is relatively loose. After 4 freeze–thaw cycles, the internal cracks of the specimen have deepened and increased in number, there are signs of coalescence between each crack, and the degree of structural looseness is serious. However, when the LS content is 12%, as shown in Figure 13c,d, the internal crevices are greatly reduced compared with the specimen group without LS, and the secondary hydration product C-S-H is attached to the inside and around the fissures, resulting in a more compact structure. After 4 freeze–thaw cycles, it can also be observed that the number of internal fissures has increased, and the depth of cracks has deepened, but the increasing degree is very small in comparison with the LC-0 group, indicating that the cementitious material generated by the secondary hydration reaction between the mineral composition of LS and the cement hydration product can effectively improve the compactness of the structure, and thus improve the frost resistance of cement-soil; this is close to the mechanism reported by Chai and Zhang [10] in the study of cement and additives improving warm and ice-rich frozen soil properties.

## 4. Performance Improvement Principle

**Figure 14:**
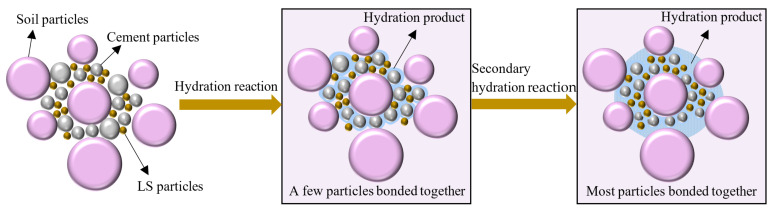
Schematic diagram of the internal action mechanism of LS on cement-soil.

The main chemical components in the Portland cement clinker used in this experiment are the four oxides CaO, ${\mathrm{SiO}}_{2}$, ${\mathrm{Al}}_{2}O_{3}$, and ${\mathrm{Fe}}_{2}O_{3}$. These oxides constitute cement minerals such as tricalcium silicate ($C_{3}S$), dicalcium silicate ($C_{2}S$), tricalcium aluminate ($C_{3}A$), and tetracalcium aluminoferrite ($C_{4}\mathrm{AF}$), respectively [46]. During the curing process of cement-soil, the cement first undergoes hydrolysis and hydration reaction under the action of the oxides to generate hydrated calcium silicate gel, calcium hydroxide, hydrated calcium aluminate, hydrated calcium ferrite, and hydrated calcium sulphoaluminate crystals [47]. Among them, tricalcium silicate, tetracalcium ferroaluminate, and tricalcium aluminate have rapid reaction rates in the hydration process of cement, which directly contribute to the early performance of cement-soil. When the solution reaches saturation, it is suspended in the solution as a gel particle, and a portion of these particles form a spatial framework through self-condensation and hardening, thereby enhancing the bond strength and integrity between cement particles [46]. The other portion and excess ${\mathrm{Ca}}^{2+}$ ionization occurs in the secondary hydration reaction with active mineral components such as ${\mathrm{SiO}}_{2}$. and ${\mathrm{Al}}_{2}O_{3}$. in LS, with $\mathrm{Ca}{\left(\mathrm{OH}\right)}_{2}$ constituting the majority of the substances involved in the reaction, as shown in Equations (2) and (3). This reaction can promote further cementation within cement-soil, and the generated cementitious materials, such as hydrated calcium silicate and hydrated calcium aluminate, wrapped and connected the soil particles [40], as shown in Figure 14.

(2) $$3{\mathrm{Ca}}^{2+}+2{\mathrm{Si}}^{4+}+14{\mathrm{OH}}^{-}\to 3\mathrm{CaO}\cdot 2{\mathrm{SiO}}_{2}\cdot 3H_{2}O+4H_{2}O$$

(3) $$4{\mathrm{Ca}}^{2+}+4{\mathrm{Al}}^{3+}+20{\mathrm{OH}}^{-}+3H_{2}O\to 3\mathrm{CaO}\cdot 2{\mathrm{Al}}_{2}O_{3}\cdot \mathrm{Ca}{\left(\mathrm{OH}\right)}_{2}\cdot 12H_{2}O$$

Figure 14 clearly shows that, in the process of cement hydration, the generated hydration products will bond a small number of soil particles, and when the mineral composition in LS participates in the secondary hydration reaction, the hydration products increase; the bond network between soil particles gradually expands; and, because of the condensation and hardening of the secondary hydration products, the strength of the particle skeleton increases, which in turn leads to a significant increase in the frost resistance of cement-soil. In addition, the improvement mechanism of LS on cement-soil after 90 days of curing is discussed in this experiment, which is consistent with the conclusion reported by Li and Huang [30] in the research finding that the incorporation of LS in concrete is more conducive to improving the internal transition interface of concrete at the later stage. Notably, Figure 14 exhibits that, as the hydration reaction progresses, the unhydrated cement particles gradually decrease [48]. When too much LS is added, the ${\mathrm{Ca}}^{2+}$ in the hydration reaction is insufficient to participate in the reaction, resulting in the decrease in hydration products. Therefore, the performance of the specimen group with an LS content of 18% in this paper is inferior in all respects to that of the test group with an LS content of 12%.

## 5. Conclusions

The purpose of this study is to explore the feasibility of LS to improve the frost resistance of cement-soil. The surface damage and mass loss, unconfined compression strength, triaxial shear strength, cohesion, and internal friction angle of cement-soil with different LS contents (0%, 6%, 12%, and 18%) under different freeze–thaw cycles (0, 2, and 4) were tested and compared. The influence mechanism of LS on cement-soil was analyzed from the microscopic point of view by NMR and SEM. The main conclusions are as follows:

1. The degree of surface damage of the cement-soil specimens with different LS contents varied after 4 freeze–thaw cycles. The incorporation of LS suppressed the propagation of freeze–thaw damage cracks on the surface of the specimens, and the damage area was also reduced. By testing the mass change in the specimens before and after freeze–thaw cycles, it is found that the mass loss values of the specimen groups incorporated with LS were less than those of the groups without LS incorporated.
2. LS can also improve the unconfined compression strength and shear strength of cement-soil. The strength values of cement-soil with LS are higher than those without LS under 0, 2, and 4 freeze–thaw cycles, and the strength value increases most obviously when the content of LS is 12%.
3. The effect of LS incorporation on the cohesion and internal friction angle of the cement-soil was also significant, in which the cohesion was the key to the improvement in the performance of the cement-soil, and the cohesion of the specimen group with 12% LS incorporation after 4 freeze–thaw cycles was increased by 1.1 MPa compared with that of the specimen group without LS incorporation.
4. The incorporation of LS effectively reduces the porosity and pore size inside the cement-soil, and the relatively high content of ${\mathrm{SiO}}_{2}$. and ${\mathrm{Al}}_{2}O_{2}$ in LS with a certain degree of pozzolanic activity can generate gels such as C-S-H by secondary hydration reaction inside the cement-soil, which helps to increase the bonding skeleton inside the cement-soil, and thus resist the crack growth under the freeze–thaw action.

This study provides ideas for improvements to the frost resistance of cement-soil and the comprehensive application of LS. However, this paper only considers the performance of cement-soil after 90 days of curing, and the maximum number of freeze–thaw cycles is set to 4. In future research work, the effect of LS content on the frost resistance of cement-soil under different curing ages and multiple freeze–thaw cycles will be investigated further. In addition, the coupling influence of chloride ion erosion and carbonation on the frost resistance of LS cement-soil will be explored in greater depth.

## Figures and Tables

**Table 1:** Basic properties of cement.

Properties	Value
Scheme 338	$338\;m^{2}\cdot {\mathrm{kg}}^{-1}$
Cement soundness	qualified
Initial setting time	195 min
Final setting time	393 min
3-day compressive strength	23.8 MPa
28-day compressive strength	37.2 MPa
3-day flexural strength	5.7 MPa
28-day flexural strength	7.2 MPa

**Table 2:** Chemical compositions of cement and LS (wt.%).

Chemical Components	Cement	Lithium Slag
${\mathrm{SiO}}_{2}$	19.17	56.26
CaO	58.54	9.83
${\mathrm{Al}}_{2}O_{3}$	7.26	17.39
${\mathrm{Fe}}_{2}O_{3}$	3.96	0.96
${\mathrm{Na}}_{2}O$	-	1.32
MgO	1.09	0.26
$K_{2}O$	-	1.21
${\mathrm{SiO}}_{3}$	5.07	5.82
${\mathrm{TiO}}_{2}$	-	0.17
Others	3.86	1.13
LOI ^a^	1.05	5.65

^a^ Loss on ignition.

**Table 3:** Basic physical indexes of soil.

Properties	Value
Natural moisture content	27.2%
Natural weight	$19.7\;\mathrm{kN}\cdot m^{-3}$
Natural density	$1.96\;g\cdot {\mathrm{cm}}^{-3}$
Liquid limit	33.5%
Plastic limit	18.7%
Liquidity index	0.56
Plasticity index	14.8

**Table 4:** Mix proportion of specimens (mass %).

Specimens	Cement	LS	Soil	Water	Water/Binder Ratio
LC-0 ^a^	12	0	88	20.4	1.7
LC-6	12	6	82	20.4	1.7
LC-12	12	12	76	20.4	1.7
LC-18	12	18	70	20.4	1.7

^a^ LC-0 means the content of lithium slag is 0%. The meanings of the other groups in the table can be deduced by analogy.

## Data Availability

The data used to support the findings of this study are available from the corresponding author upon request.
